# Genetic Diversity, Linkage Disequilibrium and Selection Signatures in Chinese and Western Pigs Revealed by Genome-Wide SNP Markers

**DOI:** 10.1371/journal.pone.0056001

**Published:** 2013-02-07

**Authors:** Huashui Ai, Lusheng Huang, Jun Ren

**Affiliations:** Key Laboratory for Animal Biotechnology of Jiangxi Province and the Ministry of Agriculture of China, Jiangxi Agricultural University, Nanchang, China; National Institute of Allergy and Infectious Diseases, United States of America

## Abstract

To investigate population structure, linkage disequilibrium (LD) pattern and selection signature at the genome level in Chinese and Western pigs, we genotyped 304 unrelated animals from 18 diverse populations using porcine 60 K SNP chips. We confirmed the divergent evolution between Chinese and Western pigs and showed distinct topological structures of the tested populations. We acquired the evidence for the introgression of Western pigs into two Chinese pig breeds. Analysis of runs of homozygosity revealed that historical inbreeding reduced genetic variability in several Chinese breeds. We found that intrapopulation LD extents are roughly comparable between Chinese and Western pigs. However, interpopulation LD is much longer in Western pigs compared with Chinese pigs with average r^2^_0.3_ values of 125 kb for Western pigs and only 10.5 kb for Chinese pigs. The finding indicates that higher-density markers are required to capture LD with causal variants in genome-wide association studies and genomic selection on Chinese pigs. Further, we looked across the genome to identify candidate loci under selection using *F_ST_* outlier tests on two contrast samples: Tibetan pigs versus lowland pigs and belted pigs against non-belted pigs. Interestingly, we highlighted several genes including *ADAMTS12*, *SIM1* and *NOS1* that show signatures of natural selection in Tibetan pigs and are likely important for genetic adaptation to high altitude. Comparison of our findings with previous reports indicates that the underlying genetic basis for high-altitude adaptation in Tibetan pigs, Tibetan peoples and yaks is likely distinct from one another. Moreover, we identified the strongest signal of directional selection at the *EDNRB* loci in Chinese belted pigs, supporting *EDNRB* as a promising candidate gene for the white belt coat color in Chinese pigs. Altogether, our findings advance the understanding of the genome biology of Chinese and Western pigs.

## Introduction

The pig is an important agriculture animal that produces large scale of animal-based protein for human consumption. It also services as an important animal model for human diseases [1]. It is thus of great interest to dissect the molecular basis of pig complex traits such as growth, energy metabolism, reproduction and behavior.

It is known that domestic pigs were originated independently from multiple subspecies of European and Asian wild boars [2]–[4]. During approximate 10,000 years of domestication, both natural (environmental pressures) and artificial (human civilization) selection have contributed to shape and stabilize distinct pig breeds that show diverse phenotypes in conformation, production and meat quality traits. For instance, Chinese indigenous breeds are known for their low growth rate, poor feed conversion efficiency but early puberty and good meat quality [5]. On the contrary, Western commercial breeds, such as Large White, Duroc, Landrace and Pietrain, have characteristics of high meat yield, fast growth rate and excellent feed efficiency but unfavoured meat quality. These diverse breeds provide ideal experimental materials for the genetic dissection of porcine complex traits.

Recently, the next-generation sequencing technology has markedly facilitated the genetic studies of complex traits in domestic animals. The novel technology has been used to reveal domestication, natural and artificial selection footprint in the porcine genome through resequencing diverse pig samples. For instance, using massive parallel sequencing of pooled DNA of 4 Western pig breeds and 1 wild boar, Amaral et al. (2011) identified genomic differentiation regions under strong selection for muscle development, growth, behavior and coat color and consequently identified candidate genes for interesting traits [6]. However, the selection footprints in Chinese pigs remain elusive.

Currently, the whole-genome resequencing is still expensive although the price is gradually reducing. It hampers the use of the technology in large sample studies. Alternatively, the genome-wide SNP genotyping technology has emerged as a powerful tool for genetic studies in a variety of domestic animals. In 2009, a genome-wide 60 K SNP array became available in pigs [7]. The array has been used to identify loci affecting qualitative and quantitative traits, reveal population structure and evolutionary history, and ascertain linkage disequilibrium (LD) extent at the genome level in pigs. For example, researchers have used the SNP panel to estimate LD levels in Finnish [8] and US pig breeds [9]. The findings provide novel insights into not only population structures but also marker-assisted selection and genomic selection programs in the tested breeds. However, to date, the LD extent across the genome in Chinese pigs remain unexplored. The objective of this study was to investigate nucleotide variability, population structure, LD extent and selection footprints in the genome of Chinese pigs in comparison with Western pigs using the porcine 60 K SNP panel.

## Materials and Methods

### Ethics Statement

All animal work was conducted according to the guidelines for the care and use of experimental animals established by the Ministry of Agriculture of China. The ethics committee of Jiangxi Agricultural University specifically approved this study.

### Animals

**Figure 1:**
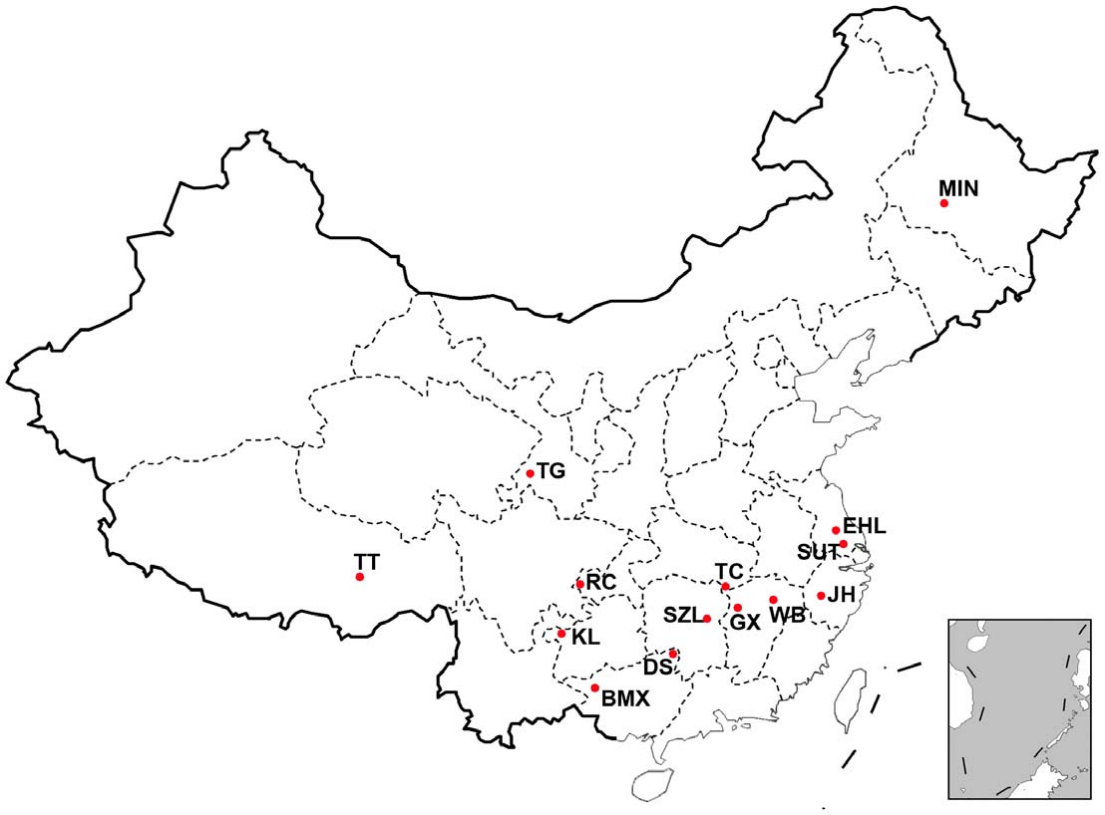
The geographic locations of Chinese pigs in the present study. BMX, Bamaxiang; DS, Dongshan; EHL, Erhualian; GX, Ganxi; JH, Jinhua; KL, Kele; MIN, Min; RC, Rongchang; SUT, Sutai; SZL, Shaziling; TC, Tongcheng; TG, Tibetan (Gansu); TT, Tibetan (Tibet); WB, Chinese wild boars.

A total of 304 unrelated individuals (no common ancestry for 3 generations) were sampled from 18 populations including 12 Chinese indigenous breeds representing 6 ecotypes, 1 Chinese cultivated breed, 1 Chinese wild boar population and 4 Western commercial breeds. Sample size per breed ranged from 5 for White Duroc to 32 for Erhualian pigs (**Table 1** **,** **Figure 1**). Genomic DNA was extracted from ear tissues using a routine phenol/chloroform way, and was diluted to a final concentration of 20 ng/µl.

**Table 1:** Sample size, genetic diversity and LD extent of 18 Chinese and Western pig populations.^a^

Population	Origin	No.	N_SNP_	Indices of Genetic Diversity			r^2^_0.3_ (kb)
				P_N_	A_R_	H_E_	
**Chinese pigs**							
Bamaxiang	Guangxi	16	13747	0.93	1.92	0.33	143
Dongshan	Guangxi	15	12342	0.84	1.83	0.29	311
Erhualian	Jiangsu	32	14184	0.97	1.96	0.34	195
Ganxi	Jiangxi	13	11292	0.76	1.75	0.27	541
Jinhua	Zhejiang	13	10755	0.76	1.74	0.26	548
Kele	Guizhou	10	15663	0.99	1.98	0.40	179
Min	Heilongjiang	15	12893	0.86	1.85	0.32	744
Rongchang	Chongqin	18	14601	0.96	1.95	0.35	83
Shaziling	Hunan	11	13237	0.88	1.88	0.33	292
Sutai	Jiangsu	15	14615	0.97	1.97	0.37	333
Tibetan	Gansu	21	13895	0.95	1.94	0.35	251
Tibetan	Tibet	29	14250	0.95	1.95	0.35	243
Tongcheng	Hubei	16	14918	0.98	1.97	0.37	101
Wild Boar	Jiangxi	15	14989	0.98	1.97	0.38	38
**Western pigs**							
Duroc	USA	20	14103	0.92	1.91	0.34	413
Landrace	Danmark	20	15028	0.97	1.96	0.38	334
Large White	Canada	20	15134	0.98	1.98	0.38	280
White Duroc	USA	5	14325	0.90	1.87	0.35	757

a N_SNP_, the number of SNPs with MAF >0.2 in the 15,911 SNP subset; P_N_, the proportion of SNP which displayed polymorphism in the 15,911 SNPs selected from the 60 K panel; A_R_, allelic richness; H_E_, expected heterozygosity. r^2^ measures were calculated between all pairs of SNPs with MAF ≥10% and <10% missing data in each population.

### Genotyping

All animals were genotyped using Porcine SNP60 BeadChips (Illumina, USA) containing 62,163 SNPs according to the manufacturer protocol. The genotypes were judged using BEADSTUDIO version 3.2 (Illumina, USA). SNPs were filtered with call rate ≥90% and minor allele frequency (MAF) ≥0.05. A total of 52,556 informative SNPs were obtained. Different subsets of SNP data were chose from the 52,556 SNPs for further statistical analyses. The SNP positions within a chromosome were based on the current pig genome assembly (Sscrofa10.2).

### Calculation of Estimates Related to Genetic Diversity and Distance

A common subset of 15,911 SNPs with MAF ≥0.2 in both Chinese and Western pigs were explored to calculate 3 measures of genetic variability: allelic richness (A_R_), the proportion of polymorphic markers (P_N_) and expected heterozygosity (H_E_). A_R_ estimates was determined using ADZE v1.0 [10]. P_N_ and H_E_ were obtained with PLINK v1.07 [11] under the default settings. All 52,556 informative SNPs were used to analyze the genetic distance between populations. The average proportion of alleles shared was calculated as Dst by PLINK v1.07 [11]:
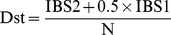



Where IBS1 and IBS2 are the number of loci which share either 1 or 2 alleles identical by state (IBS), respectively, and N is the number of loci tested. Genetic distance between all pair-wise combinations of individuals was calculated as 1-Dst. Neighbor-joining (NJ) relationship trees between individuals were constructed using Neighbor in the PHYLIP version 3.69 package [12] and were displayed by MEGA v4 [13].

### Analysis of Population Structure

Principal component analysis (PCA) was conducted using Smartpca in the EIGENSOFT version 4.0 package [14]. To avoid artifacts due to LD, we filtered the SNP data to 18700 SNPs by excluding SNPs with pair-wise genotype r^2^≥0.2. STRUCTURE [15] was employed to analyze the population structure. To save the running time and minimize SNP ascertainment bias between Chinese and Western pigs, the number of markers was reduced to 12,924 SNPs by selecting those with MAF ≥0.2 from the above-mentioned 18700 SNPs. STRUCTURE was run with 10,000 iterations using the correlated allele model. The INFERALPHA option under the admixture model was used with the allele frequency prior parameter LAMBDA set to 1. Results were plotted using DISTRUCT [16], and the results for *K* = 2 to 8 are shown.

### Determination of Genetic Differentiation Estimates

Unbiased genetic differentiation estimates of *F_ST_* [17] were calculated as described in [18] using the whole SNP data set. Briefly, *F_ST_* was estimated as follows:
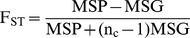



Where MSG and MSP denote the observed mean square errors for loci within and between populations respectively, n_c_ is the average sample size across samples that also incorporates and corrects for the variance in sample size over population,
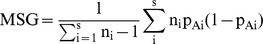


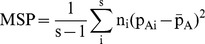


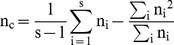



In the above formulae, n_i_ denotes the sample size in the ith population, p_Ai_ is the frequency of SNP allele A in the ith population, and 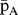 is a weighted average of p_A_ across populations. Because the range of *F_ST_* is originally defined between 0 and 1 [19], negative *F_ST_* values that do not have a biological interpretation were set to 0.

### LD Decay Assay

Pair-wise LD was calculated by the genotype correlation coefficient (r^2^). For all pairs of autosomal SNPs, r^2^ measures were calculated using the –r2–ld-window 99999–ld-window-r2 0 command in PLINK v1.07 [11]. Within each population, r^2^ measures were calculated between all pairs of SNPs with MAF ≥10% and <10% missing data. Inter-SNP distances (kb) were binned into the following classes: 0–4, 4–8, 8–12, 12–20, 20–30, 30–40, 40–60, 60–80, 80–100, 100–120, 120–160, 160–200, 200–250, 250–300, 300–360, 360–460, 460–620, 620–800, and 800–1,000 kb. Observed pair-wise LD (r^2^) was averaged for each inter-SNP distance class. To compare LD decay between Chinese and Western pigs, all 65 pigs from Western breeds and a random subset of 65 pigs from Chinese breeds were analyzed. To compare LD decay between the 18 tested populations, a random subset of 10 pigs were selected from each population, except for 5 pigs from White Duroc. The LD extent and decline between breeds were predicted by the following equation [20]–[22]

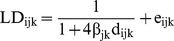
where LD_ijk_ is the observed LD for marker pair i of breed j in genomic region k, d_ijk_ is the distance in base pairs for marker pair i of breed j in genomic region k, β_jk_ is the coefficient that describes the decline of LD with distance for breed j in genomic region k, and e_ijk_ is a random residual.

### Identification of Runs of Homozygosity

Runs of homozygosity (ROHs) were identified via the Runs of Homozygosity program implemented in PLINK version 1.07 [11]. The program slides a moving window of 5 Mb (minimum 50 SNPs) across the genome to detect long contiguous runs of homozygous genotypes in each population. An occasional genotyping error or missing genotype occurring in an otherwise-unbroken homozygous segment could result in the underestimation of ROHs. To address this problem, the program allows one heterozygous and five missing calls per window. Because strong LD, typically extending up to about 100 kb, is common throughout the pig genome, short tracts of homozygosity are very prevalent. To exclude these short and very common ROHs, the minimum length for an ROH was set at 500 kb.

### Detection of SNP Loci Under Selection

BAYESCAN was employed to identify SNP outliers showing significant divergence from neutral loci, suggestive of selection [23]. The probability that a locus is under selection was estimated by calculating a Bayes factor, which is simply the ratio of the posterior probabilities of two models (selection/neutral). After 10 pilot runs of 5000 iterations each, default values of proposed distributions were updated throughout 100,000 Markov Chain Monte Carlo steps after an initial burn-in of 50,000 steps. As a result of BAYESCAN, a Bayes factor of 10 (log10 Bayes factor: 1) corresponding to a posterior probability of 0.91 was considered a “strong” evidence for selection. The top criterion of “decisive” (log10 Bayes factor greater than 2), corresponding roughly to a posterior probability range of 0.99 to 1, conclusively indicated that a locus was affected by selection.

### Characterization of Candidate Genes Under Selection

All SNP outliers filtered by BAYESCAN mapped to pig gene-associated regions by searching against the UCSC genome database (http://genome.ucsc.edu/), and human homologous genes were identified by aligning pig gene-associated regions against the human genome using the BLAST-like Alignment Tool (BLAT) [24]. A SNP was considered to be located in a gene region if it mapped to the transcription unit or located within 5 kb upstream of the initial codon or downstream of the terminal codon of target genes. To characterize candidate genes under selection, the UniProt Knowledgebase (UniProtKB) accession number of human homologs for these genes were extracted by g:Convert (http://biit.cs.ut.ee/gprofiler/gconvert.cgi) program.

## Results

### SNP Characteristics

All 304 animals were genotyped for 62,163 SNPs in the current Porcine 60 K Beadchip, of which 54,920 can be unambiguously mapped to the pig genome (Build 10.2). The SNPs were filtered with call rate ≥90% and MAF ≥0.05. A total of 52,556 informative SNPs were obtained for subsequent statistic analyses including 1113 SNPs on SSCX, 9 SNPs on SSCY and 5,859 unmapped SNPs. The average physical distance between neighboring SNPs was 55.6 kb, ranging from 45.0 kb on SSC14 to 182.0 kb on SSCY (**Table S1**). The number of informative SNPs with MAF ≥0.2 in each pig population is shown in **Table 1**.

### Genetic Diversity Across Populations

In general, Western pigs had roughly comparable SNP polymorphism with a P_N_ range from 0.90 to 0.98, compared with 0.76 to 0.98 in Chinese pigs (**Table 1**). Chinese Kele pigs displayed the highest genetic diversity as measured by allelic richness (A_R_ = 1.98) and expected heterozygosity (H_E_ = 0.40). Conversely, Chinese Ganxi and Jinhua pigs showed the lowest nucleotide variability (Jinhua: A_R_ = 1.74,, H_E_ = 0.26; Ganxi: A_R_ = 1.75, H_E_ = 0.27; **Table 1**).

### Genetic Distance within and between Populations

**Figure 2:**
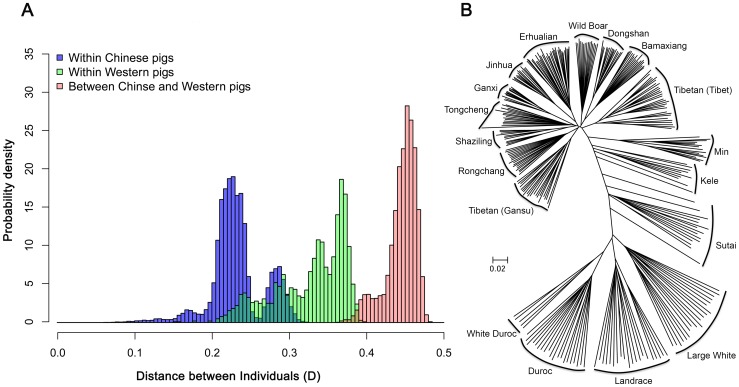
Genetic diversity within and between Chinese and Western pigs. (A) The genetic distance (D) between pairs of animals. Blue bars represent D within Chinese pigs; green bars represent D within Western pigs; red bars denote D between Chinese and Western pigs. (B) The neighbor-joining tree of the tested populations based on genome-wide allele sharing.

The average genetic distance between individuals was 0.23±0.04 within Chinese pigs, 0.33±0.05 within Western pigs and 0.45±0.02 between Chinese and Western pigs (**Figure 2A**). For each population, it ranged from 0.11±0.02 (Ganxi) to 0.23±0.04 (Kele) within Chinese pigs, in comparison with 0.24±0.01 (Duroc) to 0.28±0.01 (Large White) in Western pigs (**Figure S1**).

To examine topological relationships between individuals, a neighbor-joining tree was constructed based on genome-wide allele sharing. All individuals from the same breed gathered together. Chinese and Western pigs were clustered into two separate clades, except that Chinese Min, Kele and Sutai pigs were grouped into clades distinct from the Chinese major clade. In Western pigs, White Duroc pigs formed a cluster with Duroc pigs (**Figure 2B**).

### Population Structure of Chinese and Western Pigs

**Figure 3:**
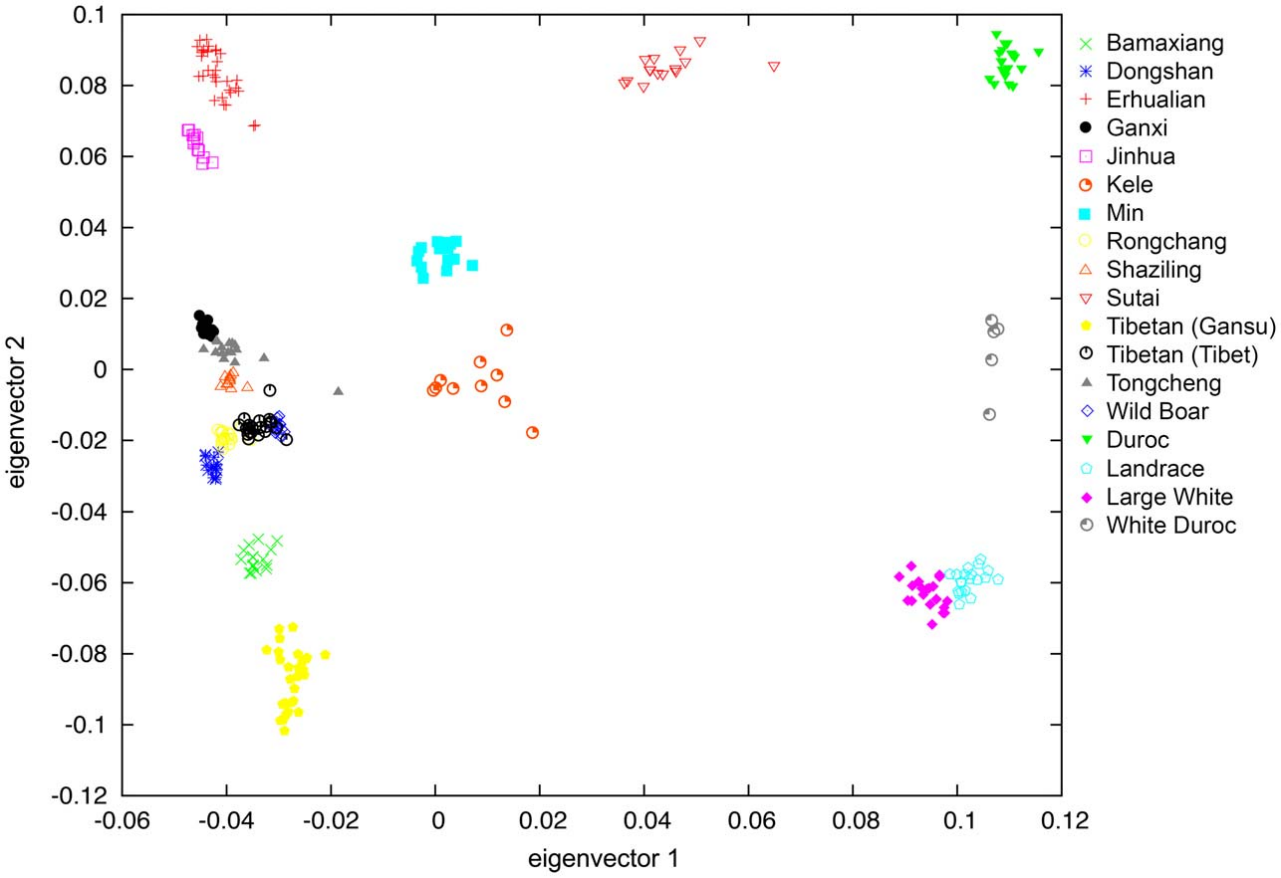
Population structures of Chinese and Western pigs revealed by principal component analysis.

We analyzed population structure of Chinese and Western pigs using PCA and the filtered genotype data of 18700 SNPs as described in Methods. The analysis clearly discriminates Chinese pigs from Western pigs on the first two PC axes. PC1 represented the Chinese versus Western pig axis, and PC2 showed the genetic difference between Western pigs (**Figure 3**). Sutai pigs were cultivated from Chinese Erhualian and Duroc pigs and were concordantly located between the two breeds. Similarly, White Duroc pigs were produced by crossing Large White with Duroc and were conceivably situated between Large White and Duroc pigs. Chinese indigenous breeds and Chinese wild boars were closely clustered. However, Chinese Min and Kele pigs were closer to Western pigs compared with Chinese pigs elsewhere. It is in good agreement with the neighbor-joining tree.

To quantify population structure and admixture patterns of the tested populations, we utilized STRUCTURE, a widely used Bayesian clustering method with a subset of SNP data (see Methods). Bayesian ancestry inference resembled the PCA results. Chinese indigenous pigs and Western commercial pigs formed the first partitions (*K* = 2), followed by Tibetan Tibet pigs (*K* = 3), Duroc pigs (*K* = 4), Dongshan and Ganxi pigs (*K* = 5), Jinhua pigs (*K* = 6), Min pigs (*K* = 7). When runs with *K* = 8, Dongshan, Ganxi, Erhualian, Tibetan (Tibet), Tibetan (Gansu), Min, Duroc and Large White or Landrace formed 8 independent populations (**Figure S2**). However, Kele pigs show consistent signals of admixture with Western pigs (**Figure S2**). From K = 3 to K = 6, Min pigs also appeared to be admixed with Western pigs.

### Population Differentiation between Chinese and Western Pigs

To determine the extent of population differentiation between Chinese and Western pigs, *F_ST_* values was estimated for all autosomal informative markers at genome-wide level. The empirical genome-wide distribution of *F_ST_* between population was determined (**Figure S3**). The *F_ST_* estimates varied along chromosomes (**Figure S3**). Many *F_ST_* values were significantly different from 0 (p-value <0.05), displaying an overall mean of 0.195 with a standard deviation of 0.251. The *F_ST_* values for all pair-wise population comparisons are shown in **Table S2**. Duroc pigs displayed the highest *F_ST_* value compared with the other populations and White Duroc had the largest divergence with Jinhua pigs. Population differentiation between Chinese and Western pigs is high with an *F_ST_* value of 0.321±0.053, in comparison with 0.117±0.031 within Chinese pigs and 0.128±0.038 within Western pigs.

### Linkage Disequilibrium and Autozygosity Segments

**Figure 4:**
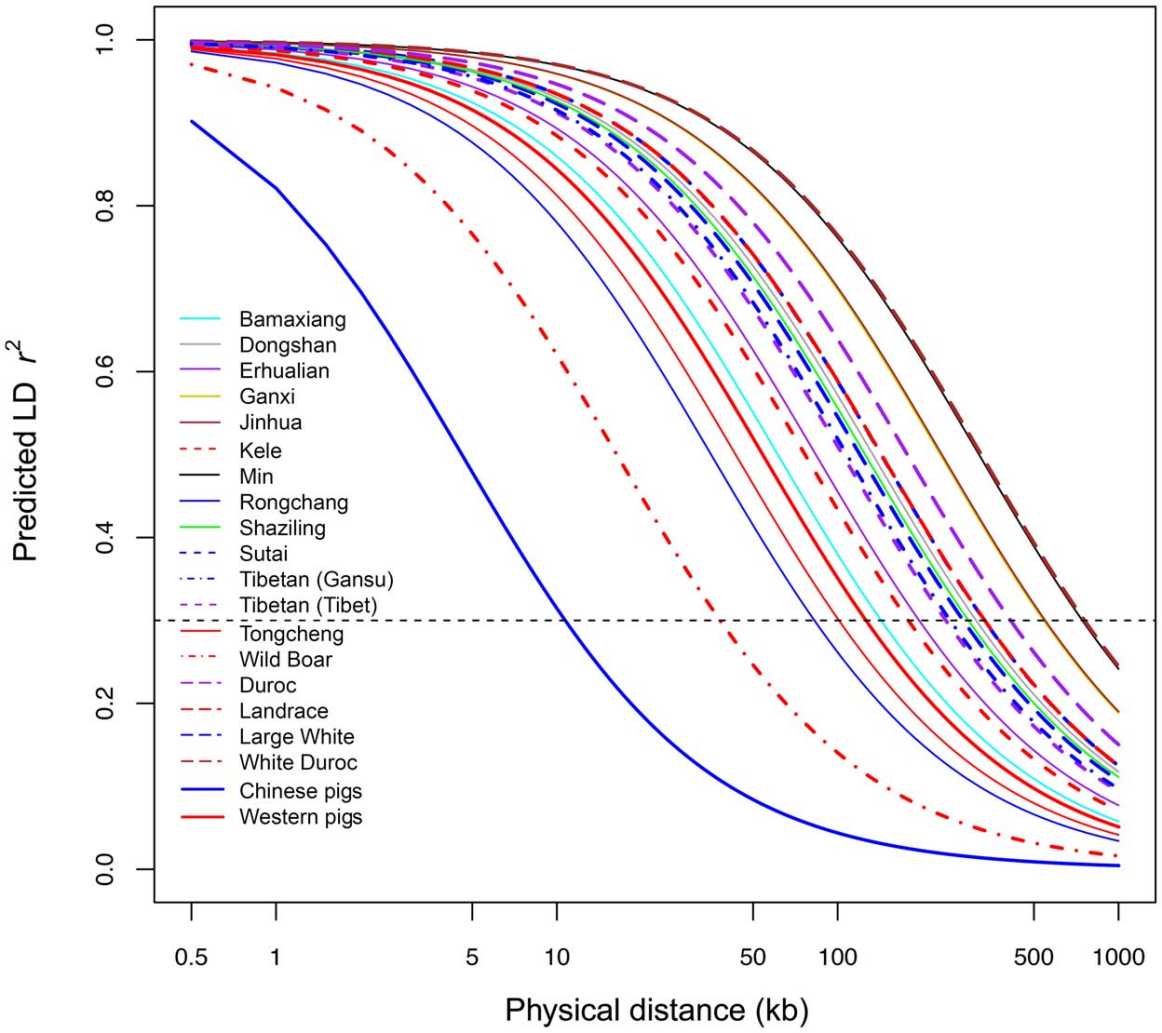
Extent of LD (predicted r^2^) as a function of inter-SNP distance between Chinese and Western pigs and within each population.

Linkage disequilibrium (r^2^) measures were calculated between all pairs of SNPs with MAF >10% and <10% missing data within each population. The number of SNP pairs ranged from 121,622 to 652,556 in these populations, and the number of SNP pairs for Chinese and Western pigs were 366,515 and 761,900, respectively. To assess LD extent patterns, we estimated r^2^_0.3_, the physical distance at which the pair-wise genotypic association in the filtered SNP data set decays below a threshold of 0.3. The LD values (r_E_^2^_0.3_) ranged from 83 kb (Rongchang) to 744 kb (Min) in Chinese pigs, and form 280 kb (Large White) to 757 kb (White Duroc) in Western pigs (**Table 1**). Chinese Wild boars had the lowest level of LD (r_E_^2^_0.3_ = 38 kb) as expected for an outbred and non-admixed population. In contrast, LD was greatest in White Duroc pigs (r_E_^2^_0.3_>750 kb), as White Duroc was a recently admixed population crossed by Duroc and Large White. Notably, the inter-population LD extent across Chinese pigs (r_E_^2^_0.3_ = 10.5 kb) is much lower than that across Western pigs (r_E_^2^_0.3_ = 125 kb) (**Figure 4**).

**Figure 5:**
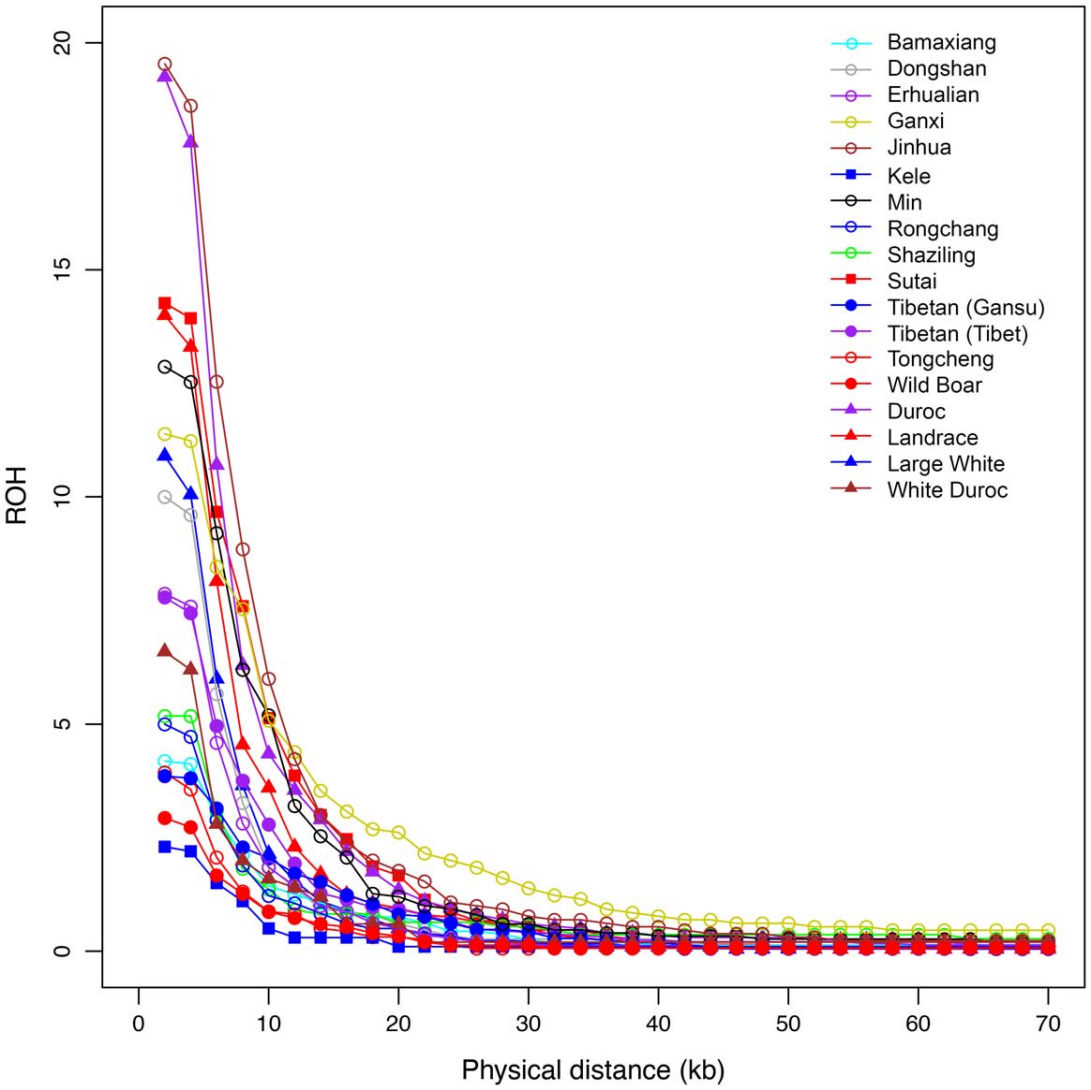
Autozygosity frequency distribution of runs of homozygosity (ROH) in Chinese and Western pig populations.

To detect the inbreeding effect on the pig genome, we examined genome-wide autozygosities that directly measures recent inbreeding. We assessed autozygosity as runs of homozygosity (ROH), and expected proportionally longer ROH and larger LD in recently inbred populations. Jinhua pigs had the highest fraction of ROH and large LD extent (r_E_^2^_0.3_ = 548 kb), suggesting recent inbreeding in the breed (**Figure 5**). It is most likely the small effective population size that causes inbreeding and large LD in Jinhua pigs. As expected, Chinese pigs with short LD extent, such as wild boars, Rongchang, Tongcheng, Bamaxiang and Tibetan pigs, always exhibit low ROH (**Figure 5**). To our surprise, Chinese Kele pigs had the shortest ROH with an even lower fraction than wild boars (**Figure 5**). We speculated that Kele pigs could have a historical admixture with Western pigs leading to short ROH. The hypothesis is supported by the above-mentioned PCA, neighbor-joining and structure analyses, where Kele pigs show close phylogenic relationships and signals of admixture with Western pigs. The introgression of Western pigs into Kele pigs should occur historically rather than recently as the breed has short-range intrapopulation LD with r^2^_0.3_ of only 179 kb. In contrast, White Duroc had the lowest fraction of autozygous segments in Western pigs (**Figure 5**) but showed the highest r^2^_0.3_ in all tested populations. This must be caused by the recent admixture of Duroc and Large White in the population. The recent admixture leads to the long-range LD extent and simultaneously reduces the length of homozygous segment in the genome.

### Candidate Genes Under Selection

**Figure 6:**
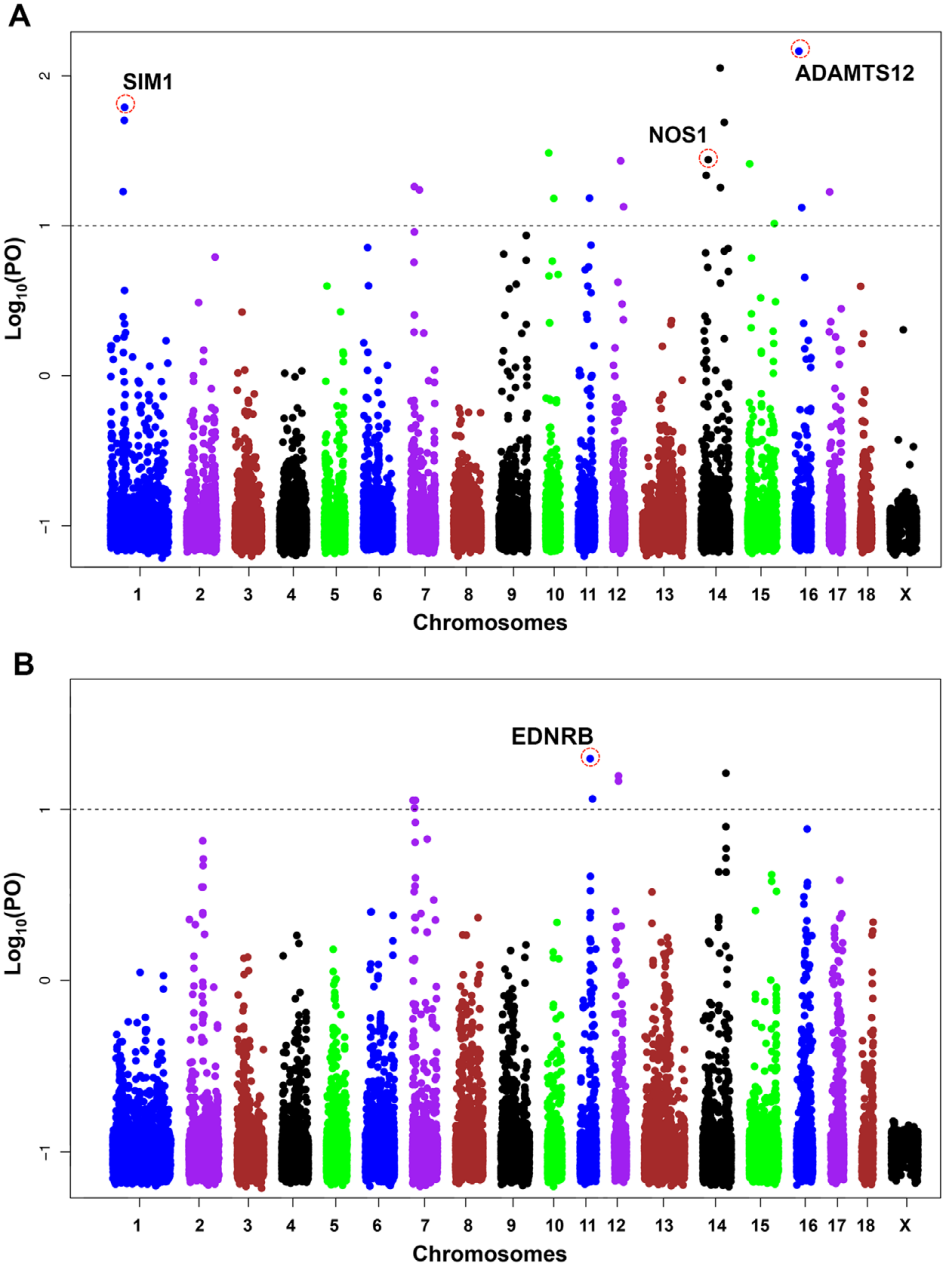
Genome-wide distribution of log_10_ Bayes factor values in two contrasts. (A) Tibetan pigs versus non-plateau pigs. (B) Belted pigs against non-belted pigs. The chromosomes are plotted along the x-axis, and log_10_ Bayes factor values are plotted along the y-axis. Chromosomes are indicated by different colors, and the threshold indicating signature of selection is denoted with a dashed grey line. In the upper panel, three highlighted genes that are likely involved with Tibetan high-altitude adaptation are circled in red, and the gene names are labeled above. For the contrast analysis between belted pigs and non-belted pigs, the top signal was detected at the *EDNRB* locus that is indicated in the lower panel.

To detect selection signatures associated with specific phenotypes, we performed the Bayescan analysis on two contrast models: plateau pigs (Tibetan) vs. non-plateau pigs and belted pigs against non-belted pigs. Two Tibetan pig populations sampled in this study are located at highland regions of 2,500–3,500 meters above sea level, while all non-plateau pigs live in lowland regions of <1,500 meters above sea level with a mean altitude value of approximate 300 meters. The sharp contrast enabled us to identify interesting genes under directional selection for adaptation to high altitude in Tibetan pigs. We identified 20 SNP outliers suggesting “strong” selection in these samples. The SNP outliers correspond to 13 candidate genes on 7 chromosomes (**Table S3**). Of the 13 genes, *ADAMTS12* and SIM1 are the two strongest loci (**Figure 6A**). In the analysis of belted pigs against non-belted pigs, 7 Chinese belted pig populations comprising Bamaxiang, Dongshan, Ganxi, Jinhua, Rongchang, Shaziling and Tongcheng were treated as one group and the other 11 populations defined the contrast group. The belted pigs show the unique two-end-black coat color phenotype with black heads and hips and white belt across bodies. The appearance is distinct from those of non-belted pigs. We looked across the genome to identify regions showing evidence of positive selection in Chinese belted pigs. A total of 8 SNP outliers on 4 chromosomes achieved the significance threshold (**Table S4**). The most significant selection signature was observed at the *EDNRB* locus on chromosome 11 (**Figure 6B**).

## Discussion

### Genetic Diversity within Chinese Indigenous Pigs

Chinese indigenous pigs are much older domesticated animals compared with Western modern pigs. Historically, Chinese pigs have not undergone intensive selection as experienced in Western pigs. Hence, it is reasonable to assume that Chinese pigs sustain higher levels of genetic variability than Western pigs. The assumption is supported by previous reports on the basis of microsatellite markers [25]–[28]. In this study, we assessed nucleotide variability using high-density SNP genotyping arrays. The SNPs on swine 60 K arrays were primarily ascertained from comparison of a small panel of Western pig genomes [7]. Given the genetic divergence between Chinese and Western pigs, we thus predicted and then observed an ascertainment bias of increasing monomorphism in Chinese pigs. A total of 15101±6625 monomorphic SNPs were detected in Chinese pigs in contrast to 5925±6625 noninformative SNPs in Western pigs. To reduce the severity of this bias, we used a more common subset of SNPs with MAF ≥0.2 (approximate 15,000) to evaluate genetic diversity in Chinese and Western pigs. We found that Chinese pigs had roughly comparable SNP polymorphisms to Western pigs. Several Chinese breeds, such as Ganxi, Jinhua and Dongshan, showed even lower levels of variability than Western pigs. The reasons for the inconsistency with our expectation could be the current small population effective sizes of Chinese pigs and the under-representativeness of our samples. During the past decades Western commercial breeds, like Large White, Landrace and Duroc, have been widely used and dominated in Chinese pig industry. Population sizes of Chinese indigenous breeds are reducing dramatically. At present, only a limited number of pigs from each breed are raised in state-owned conservation farms. Small population sizes probably lead to decreased genetic diversity at least in some Chinese breeds. On the other way, we can not exclude the possibility that our samples under-represent genomic pool of Chinese pigs although we collected a number of unrelated animals to cover a broad consanguinity for each population.

### Genetic and Population Structure among Chinese and Western Pigs

It has been repeatedly evidenced that European and Chinese pigs have diverged from each other and have originated independently from different subspecies of ancestral wild boars approximately 8,000–10,000 years ago [2], [4], [27], [29]–[31]. Here we used the Illumina PorcineSNP60 chips to analyze the population structure between Western and Chinese pigs. In both PCA and NJ analyses, Western pigs clustered together and Chinese pigs define separate groupings except for Sutai, Min and Kele pigs. Sutai pigs were produced by crossing Chinese Taihu pigs with Western Duroc pigs. It was hence conceivable that Sutai pigs form a separate clade between Chinese and Western major clusters. Chinese Kele and Min pigs unexpectedly form branches distinct from the other Chinese indigenous pigs. It is likely caused by a historical introgression of Western pigs into the two breeds. The hypothesis is supported by the STRUCTURE analysis that revealed the signal of admixture with Western pigs in the two breeds.

In the NJ tree, Chinese pigs that have neighboring geographical locations were usually grouped together. For example, Shaziling, Tongcheng and Ganxi pigs that distribute in a boundary region of Hubei, Hunan and Jiangxi provinces were clustered into the same sub-branch. Two highly prolific breeds of Erhualian and Jinhua pigs belonging to the Lower Yangtze River Basin type were group into a clade. Moreover, two belted pigs from Guangxi province (Bamaxiang and Dongshan) form the same group. These observations are not unexpected considering that breeds of geographically close origin could more likely share common ancestors or cross to each other.

### LD Extent in Chinese Pigs

Information about genome-wide LD extent and decay is essential for GWAS mapping of loci affecting economically important traits and the implantation of genomic selection in farm animals. Previous studies have shown that LD extent is much higher in Western commercial pigs [8], [9] compared with human populations. We herein used the Porcine60k chip to generate high-density SNP genotypes on a sample of individuals representing Chinese and Western pigs. We found that Western pigs and many Chinese pigs have long-range LD within populations. It is known that the LD extent in a population depends on the history of the population, especially its effective population size. Western present-day commercial pigs have undergone strong artificial selection for lean production in the past decades, resulting in small effective population sizes. For instance, the current effective population sizes of Finnish Landrace and Large White (Yorkshire) are estimated to be only 91 and 61, respectively [8]. On the other hand, most of Chinese pigs are raised in state-run conservation farms with limited boars and a number of sows (approximately 10 boars and 100 sows in most cases). They also should have small effective population sizes although strong artificial selection has not been implemented on these pigs. It is thus conceivable to observe the roughly comparable LD extent within populations between Chinese and Western pigs. Chinese Ganxi, Jinhua and Min pigs even show higher LD extent than Western pigs. This is likely caused by smaller effective population sizes of these breeds.

Of note, the LD extent across populations is much shorter in Chinese pigs than Western pigs. With a threshold of *r* ^2^ = 0.3, LD extends to only 10.5 kb across Chinese pigs in stark contrast to 125 kb across Western pigs. The finding is comparable to Amaral et al’s report that was based on 371 SNP data in 3 genomic regions [22]. We know that Chinese pigs are much older domesticated animals compared with Western modern breeds like Large White, Landrace and Duroc. Much more generations of recombination lead to shorter identical-by-descent chromosome segments across Chinese pigs. Consequently, Chinese pigs have shorter interpopulation LD than Western pigs. Dogs and cattle have similar LD patterns to Chinese pigs with the long-range LD within populations and short-long range LD across populations [32].

Our findings of LD patterns have the following important implications. First, it would be efficient to identify responsible genes for monogenic traits segregating in multiple Chinese breeds by a two-stage mapping strategy that was originally used in dogs [33]. We can first map the locus within a single breed using a relatively sparse marker set across the genome and a few animals, and then refine the locus using a dense set of markers in a number of animals from multiple breeds. With this strategy, we have successfully identified the causal mutation for the brown coat color phenotype in Chinese pigs [34]. Second, higher mapping resolution is expected for GWAS on Chinese pigs like Rongchang and Bamaxiang that show shorter-range LD than Western pigs. However, higher dense markers than the current 60 K chip are required to capture LD with causal variants affecting phenotypic traits in these Chinese breeds. Third, genomic selection could be likely limited for Chinese pigs, especially when applied for multiple populations, considering the low persistence of LD phase across populations and the expensive cost of high-density panels for SNP genotyping.

### Candidate Genes Under Selection in the Pig Genome

It is well known that natural and artificial selection has shaped dramatically the pig genomic variability during the process of pig domestication and breeding. We utilize filtered SNP dataset (MAF>0.05, SNP calling >0.90) of Porcine60k Beadchip to detect selection signatures in the pig genome by the Bayesian likelihood method. We focused on the BayeScan analyses of two contrast models: Tibetan pigs vs. non-plateau pigs and belted pigs against non-belted pigs. The analyses allowed us to specifically identify candidate genes under directional selection for high-altitude adaptation and the white belt coat colors in Chinese pigs.

Tibetan pigs have been raised in the Qinghai-Tibetan Plateau for a long period. The long-term residence in the Plateau leads to the unique characteristics of high-altitude (>2,500 meters) adaptation in Tibetan pigs. However, the genetic determinant of the adaptation remains unknown. We performed the Bayesian scan to identified genomic regions implicated in Tibetan high-altitude adaptation using the 60 k dense SNP data. We identified 13 candidate genes showing evidence of natural selection on two Tibetan pig populations. Most of these genes have not been identified in previous human and yak studies of high-altitude samples [35]–[37]. However, three candidate genes, *ADAMTS12*, *SIM1* and *NOS1* have potential function with respect to altitude phenotypes. In mice, *SIM1* is involved with response to the hypoxia (a deficiency of available oxygen) treatment resembling the high-altitude environment [38], and nitric oxide generated by *NOS1* is an important physiological modulator of respiration during hypoxia [39]. In humans, *ADAMTS12* modulates the Ras pathway, which is implicated in oxygen sensing and metabolism [40]. It is hence likely that *ADAMTS12*, *SIM1* and *NOS1* play important roles in high-altitude adaptation in Tibetan pigs. We did not identify signatures of natural selection in several genes including *EPAS1*, *EGLN1*, *PPARA*, *ADAM17*, *ARG2*, *Mmp3* previously known to be important in Tibetan and yak hypoxic adaptation [35]–[37]. These results suggest that the underlying genetic basis for the adaptation the same highland environment in Tibetans, Tibetan pigs and yak is distinct from one another. The findings provide a basis for further investigation to confirm the role of these highlighted candidate genes in adaptation to high altitude, and would ultimately benefit studies of hypoxia-related diseases in humans.

Artificial selection is required for the formation of breed characteristics in domestic animals. It is well-known that coat colors are important breed characteristics and have undergone strong artificial selection in domestic pigs. Chinese belted pigs including Bamaxiang, Dongshan, Ganxi, Tongcheng and Shaziling have the unique two-end-black coat color phenotype. They uniformly show black heads and hips and white belt across bodies. To date, the molecular basis of the white belt phenotype remains unexplored. We performed the contrast analysis of the belted pigs versus non-belted pigs and identified 8 SNP outliers across the genome. The top signal of directional selection was evidenced at the *EDNRB* locus. *EDNRB* is a well-characterized gene contributing to coat color phenotypes in mammals. It has been shown that *EDNRB* deficient mice and horse exhibit white belt coat colors, resembling the two-end-black phenotype in Chinese pigs [41]–[42]. Therefore, our finding strongly support that *EDNRB* is a promising candidate gene for the white belt coat color in Chinese pigs. Further investigation of the *EDNRB* gene is warranted to elucidate the underlying molecular mechanism of the coat color phenotype of Chinese belted pigs, which would provide novel insights into pigmentation in mammals.

### Conclusions

We made a comprehensive survey of nucleotide variability across the porcine genome in Chinese and Western pigs. We confirmed the divergent evolution and distinct population structure between Chinese and Western pigs. For the first time, we revealed the LD extent at the genome level in Chinese pigs, and showed the effect of inbreeding and admixture on LD. Moreover, we identified promising candidate genes under directional selection for high-altitude adaptation in Tibetan pigs and the two-end-black coat color in Chinese belted pigs. The findings improve our understanding of the genome biology of Chinese and Western pigs.

## Supporting Information

Figure S1 **The genetic distance between pair-wise individuals within each pig population.** BMX, Bamaxiang; DS, Dongshan; EHL, Erhualian; GX, Ganxi; JH, Jinhua; KL, Kele; MIN, Min; RC, Rongchang; SUT, Sutai; SZL, Shaziling; TC, Tongcheng; TG, Tibetan (Gansu); TT, Tibetan (Tibet); WB, Chinese wild boars; DRC, Duroc; LR, Landrace; LW, Large White; WD, White Duroc.
(TIF)

Figure S2 **Population structure of Chinese and Western pigs revealed by the STRUCTURE software.** The abbreviated name of each population is the same as those shown in the legend of Figure S1.
(TIF)

Figure S3 ***F_ST_*** **distribution between Chinese and Western pigs.** Lower triangle: the empirical genome-wide distribution of *F_ST_*; Upper triangle: *F_ST_* distribution along different chromosomes. The abbreviated name of each population is the same as those shown in the legend of Figure S1.
(TIF)

Table S1 **Distribution of SNPs in the porcine genome.**
(DOC)

Table S2 **Genetic differentiation (** ***F_ST_*** **values) between 18 pig populations.**
(DOC)

Table S3 **SNP outliers and candidate genes under selection identified by BAYESCAN in the contrast of plateau (Tibetan) pigs vs. non-plateau pigs.**
(XLS)

Table S4 **SNP outliers and candidate genes under selection identified by BAYESCAN in the contrast of belted pigs vs. non-belted pigs.**
(XLS)
