# Supplementary figures and images for: Genetic Diversity, Linkage Disequilibrium and Selection Signatures in Chinese and Western Pigs Revealed by Genome-Wide SNP Markers
Source: PLoS One. 2013 Feb 7;8(2):e56001. doi: 10.1371/journal.pone.0056001 (PMC3567019; doi:10.1371/journal.pone.0056001)

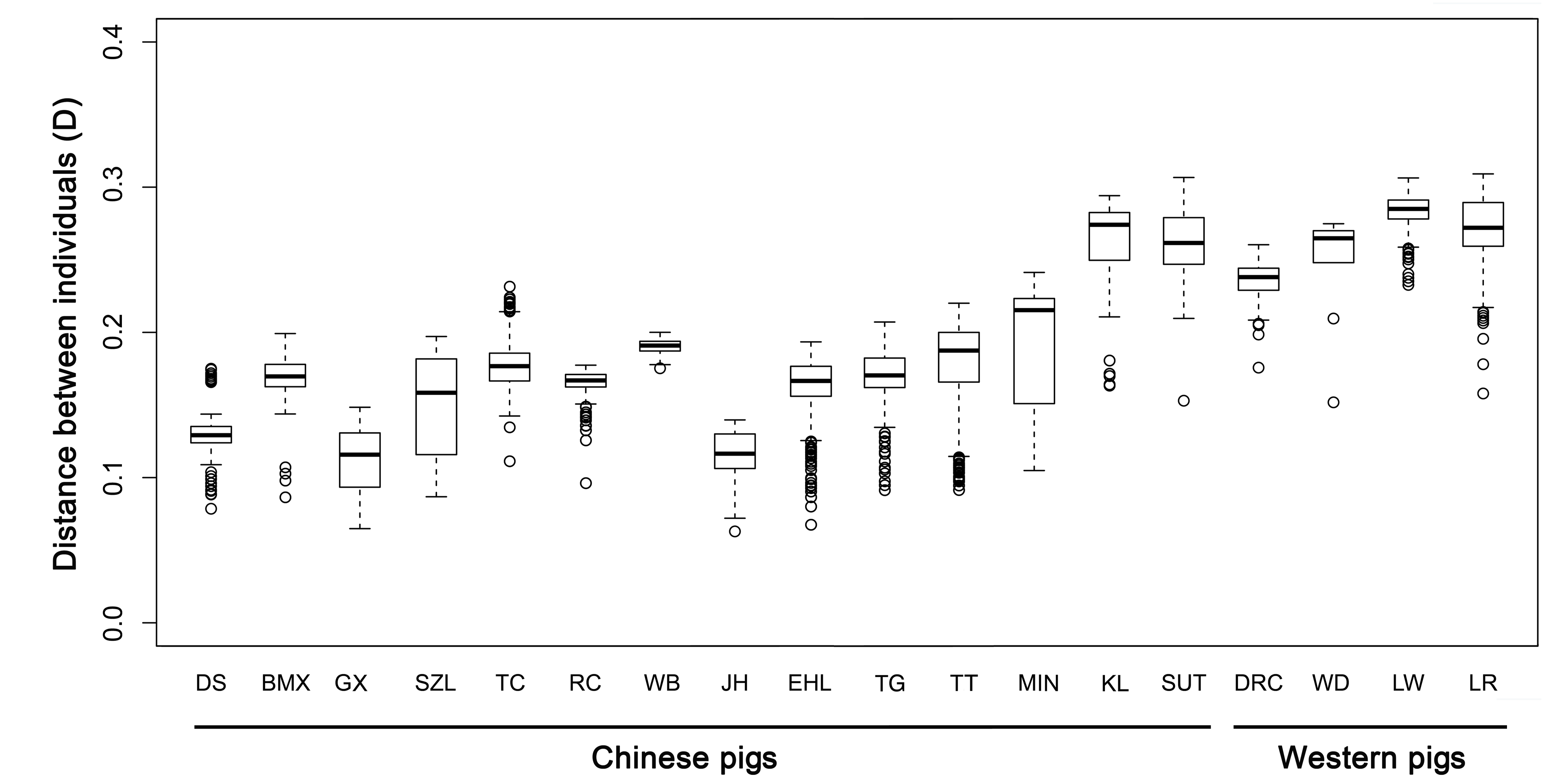

Supplement: Figure S1 — The genetic distance between pair-wise individuals within each pig population. BMX, Bamaxiang; DS, Dongshan; EHL, Erhualian; GX, Ganxi; JH, Jinhua; KL, Kele; MIN, Min; RC, Rongchang; SUT, Sutai; SZL, Shaziling; TC, Tongcheng; TG, Tibetan (Gansu); TT, Tibetan (Tibet); WB, Chinese wild boars; DRC, Duroc; LR, Landrace; LW, Large White; WD, White Duroc. (TIF) [file pone.0056001.s001.tif]

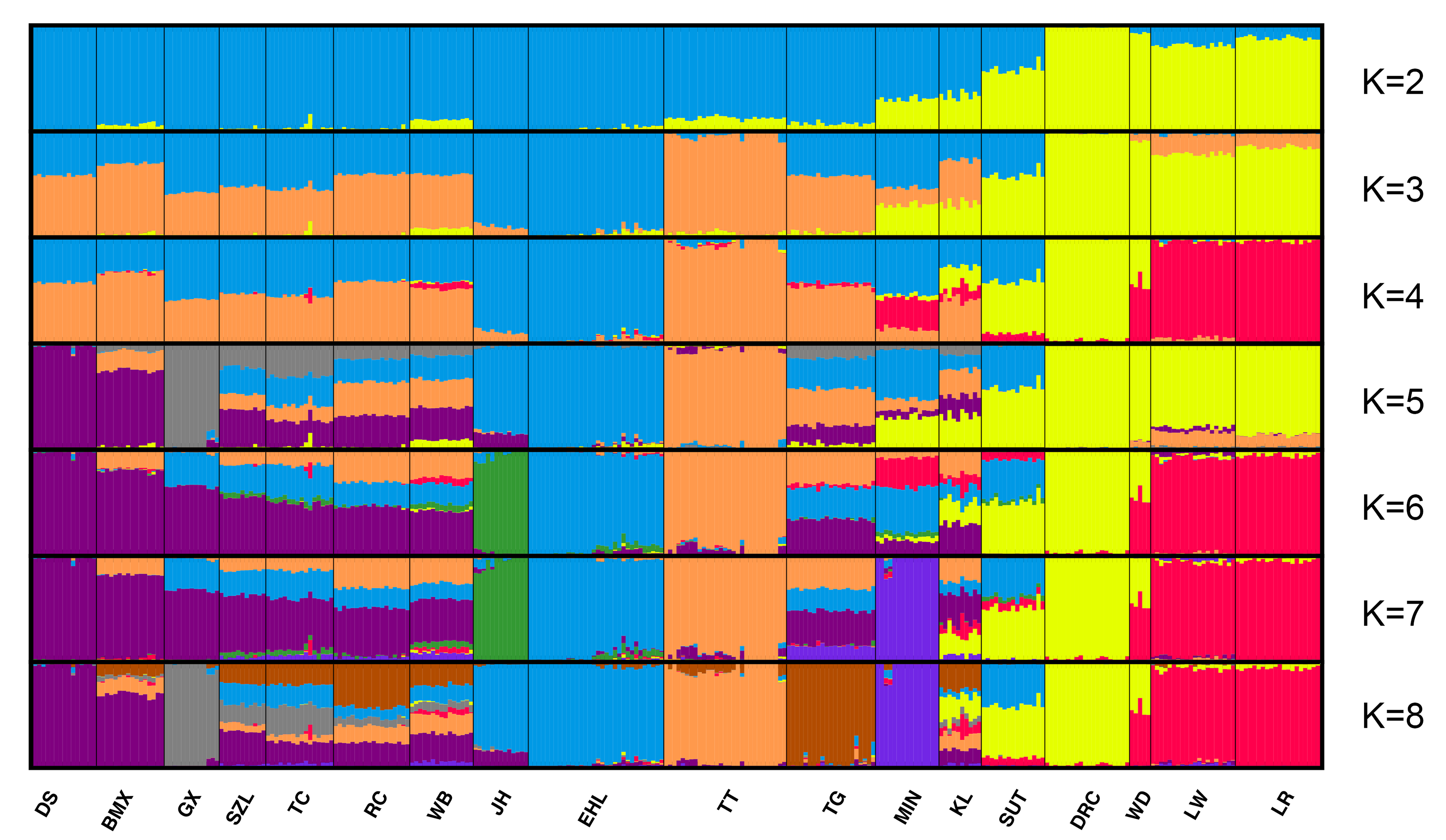

Supplement: Figure S2 — Population structure of Chinese and Western pigs revealed by the STRUCTURE software. The abbreviated name of each population is the same as those shown in the legend of Figure S1. (TIF) [file pone.0056001.s002.tif]

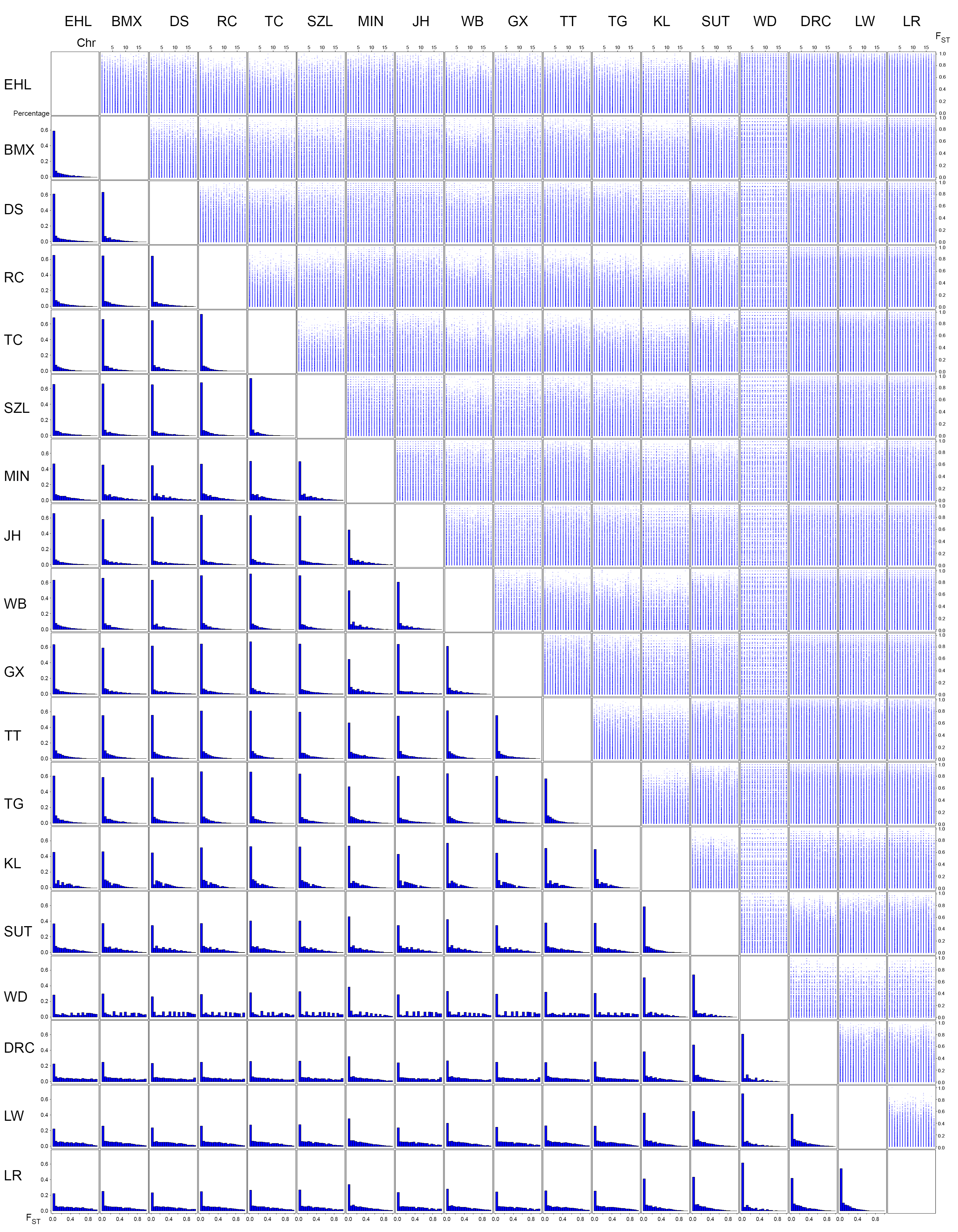

Supplement: Figure S3 — FST distribution between Chinese and Western pigs. Lower triangle: the empirical genome-wide distribution of FST; Upper triangle: FST distribution along different chromosomes. The abbreviated name of each population is the same as those shown in the legend of Figure S1. (TIF) [file pone.0056001.s003.tif]
