# Supplementary material for: Genetic Diversity, Linkage Disequilibrium and Selection Signatures in Chinese and Western Pigs Revealed by Genome-Wide SNP Markers
Source: PLoS One. 2013 Feb 7;8(2):e56001. doi: 10.1371/journal.pone.0056001 (PMC3567019; doi:10.1371/journal.pone.0056001)
Supplement: Table S1 — Distribution of SNPs in the porcine genome. (DOC) [file pone.0056001.s004.doc]

**Table S1** Distribution of SNPs in the porcine genome.

| SSC | Physical size (Mb) | SNP no. | Kb/SNP | SNP no. (after filtered) | Kb/SNP (after filtered) |
| --- | --- | --- | --- | --- | --- |
| 1 | 315.3 | 6733 | 46.8 | 5627 | 56.0 |
| 2 | 162.6 | 3444 | 47.2 | 2910 | 55.9 |
| 3 | 144.8 | 2877 | 50.3 | 2469 | 58.6 |
| 4 | 143.5 | 3664 | 39.2 | 3129 | 45.9 |
| 5 | 111.5 | 2436 | 45.8 | 2055 | 54.3 |
| 6 | 157.8 | 3244 | 48.6 | 2808 | 56.2 |
| 7 | 134.8 | 3437 | 39.2 | 2919 | 46.2 |
| 8 | 148.5 | 2837 | 52.3 | 2461 | 60.3 |
| 9 | 153.7 | 3307 | 46.5 | 2844 | 54.0 |
| 10 | 79.1 | 1800 | 43.9 | 1544 | 51.2 |
| 11 | 87.7 | 1954 | 44.9 | 1696 | 51.7 |
| 12 | 63.6 | 1586 | 40.1 | 1392 | 45.7 |
| 13 | 218.6 | 4162 | 52.5 | 3492 | 62.6 |
| 14 | 153.9 | 3973 | 38.7 | 3419 | 45.0 |
| 15 | 157.7 | 2968 | 53.1 | 2514 | 62.7 |
| 16 | 86.9 | 1917 | 45.3 | 1649 | 52.7 |
| 17 | 69.7 | 1737 | 40.1 | 1484 | 47.0 |
| 18 | 61.2 | 1365 | 44.8 | 1163 | 52.6 |
| X | 144.3 | 1467 | 98.4 | 1113 | 129.6 |
| Y | 1.6 | 12 | 136.5 | 9 | 182.0 |
| Unmapped | - | 7243 | - | 5859 | - |
| Sum | 2597 | 62163 | 47.3* | 52556 | 55.6* |

^*^ indicates the mean distance of SNPs in pig chromosomes
