# Supplementary material for: Genetic Diversity, Linkage Disequilibrium and Selection Signatures in Chinese and Western Pigs Revealed by Genome-Wide SNP Markers
Source: PLoS One. 2013 Feb 7;8(2):e56001. doi: 10.1371/journal.pone.0056001 (PMC3567019; doi:10.1371/journal.pone.0056001)
Supplement: Table S2 — Genetic differentiation ( FST values) between 18 pig populations. (DOC) [file pone.0056001.s005.doc]

Table S2 Genetic differentiation (F_ST_ values) between 18 pig populations.

|  | EHL | BMX | DS | RC | TC | SZL | MIN | JH | WB | GX | TT | TG | KL | SUT | WD | DRC | LW |
| --- | --- | --- | --- | --- | --- | --- | --- | --- | --- | --- | --- | --- | --- | --- | --- | --- | --- |
| EHL |  |  |  |  |  |  |  |  |  |  |  |  |  |  |  |  |  |
| BMX | 0.119 |  |  |  |  |  |  |  |  |  |  |  |  |  |  |  |  |
| DS | 0.117 | 0.100 |  |  |  |  |  |  |  |  |  |  |  |  |  |  |  |
| RC | 0.087 | 0.092 | 0.103 |  |  |  |  |  |  |  |  |  |  |  |  |  |  |
| TC | 0.076 | 0.085 | 0.096 | 0.061 |  |  |  |  |  |  |  |  |  |  |  |  |  |
| SZL | 0.095 | 0.092 | 0.106 | 0.078 | 0.060 |  |  |  |  |  |  |  |  |  |  |  |  |
| MIN | 0.167 | 0.177 | 0.189 | 0.161 | 0.144 | 0.158 |  |  |  |  |  |  |  |  |  |  |  |
| JH | 0.098 | 0.136 | 0.138 | 0.110 | 0.101 | 0.119 | 0.184 |  |  |  |  |  |  |  |  |  |  |
| WB | 0.094 | 0.086 | 0.103 | 0.072 | 0.066 | 0.079 | 0.146 | 0.113 |  |  |  |  |  |  |  |  |  |
| GX | 0.109 | 0.127 | 0.132 | 0.104 | 0.081 | 0.108 | 0.184 | 0.136 | 0.111 |  |  |  |  |  |  |  |  |
| TT | 0.117 | 0.118 | 0.130 | 0.092 | 0.096 | 0.110 | 0.170 | 0.136 | 0.093 | 0.130 |  |  |  |  |  |  |  |
| TG | 0.098 | 0.113 | 0.122 | 0.078 | 0.079 | 0.096 | 0.159 | 0.121 | 0.087 | 0.119 | 0.103 |  |  |  |  |  |  |
| KL | 0.168 | 0.137 | 0.164 | 0.116 | 0.108 | 0.119 | 0.127 | 0.172 | 0.106 | 0.163 | 0.140 | 0.130 |  |  |  |  |  |
| SUT | 0.219 | 0.218 | 0.237 | 0.213 | 0.192 | 0.198 | 0.166 | 0.230 | 0.185 | 0.232 | 0.219 | 0.211 | 0.103 |  |  |  |  |
| WD | 0.403 | 0.362 | 0.400 | 0.373 | 0.345 | 0.351 | 0.264 | 0.407 | 0.326 | 0.400 | 0.356 | 0.361 | 0.178 | 0.142 |  |  |  |
| DRC | 0.369 | 0.347 | 0.367 | 0.350 | 0.337 | 0.344 | 0.277 | 0.371 | 0.324 | 0.366 | 0.339 | 0.343 | 0.221 | 0.152 | 0.116 |  |  |
| LW | 0.336 | 0.302 | 0.327 | 0.308 | 0.290 | 0.293 | 0.227 | 0.331 | 0.276 | 0.324 | 0.297 | 0.300 | 0.183 | 0.163 | 0.084 | 0.177 |  |
| LR | 0.349 | 0.321 | 0.343 | 0.324 | 0.307 | 0.311 | 0.246 | 0.345 | 0.292 | 0.339 | 0.313 | 0.314 | 0.196 | 0.172 | 0.113 | 0.174 | 0.105 |

Note: EHL, Erhualian; BMX, Bamaxiang; DS, Dongshan; RC, Rongchang; TC, Tongcheng; SZL, Shaziling; MIN, Min; JH, Jinhua; WB, Wild boars; GX, Ganxi; TT, Tibetan pigs (Tibet); TG, Tibetan pigs (Gansu); KL, Kele; SUT, Sutai; WD, White Duroc; DRC, Duroc; LW, Large White; LR, Landrace.
